# Mental Health, Quality of Life and Violence Exposure in Low-Socioeconomic Status Children and Adolescents of Guatemala

**DOI:** 10.3390/ijerph17207620

**Published:** 2020-10-19

**Authors:** Rosalba Company-Córdoba, Diego Gómez-Baya, Francisca López-Gaviño, Joaquín A. Ibáñez-Alfonso

**Affiliations:** 1 Human Neuroscience Lab, Department of Psychology, Universidad Loyola Andalucía, 41704 Sevilla, Spain; rcompany@uloyola.es; 2 Department of Social, Developmental and Educational Psychology, Universidad De Huelva, 21071 Huelva, Spain; diego.gomez@dpee.uhu.es; 3 ETEA Foundation, Development Institute of Universidad Loyola Andalucía, 14004 Córdoba, Spain; paquilopez@cop.es

**Keywords:** mental health, depression, anxiety, quality of life, violence, food insecurity, socioeconomic status, poverty, children, adolescents

## Abstract

Growing up in vulnerable conditions has an impact on children and adolescents’ mental health and well-being outcomes. However, this evidence has rarely been obtained in middle and low-income countries like Guatemala, where food insecurity and exposure to violence frequently threaten childhood development. The aim of this study was to analyse the relations that sociodemographic and socioeconomic factors have with psychological adjustment of low-socioeconomic status (SES) Guatemalan children and adolescents, and how these relations were mediated by food insecurity and exposure to violence. A total of 185 participants (50.8% girls; aged between 6 to 17, M = 11.82, SD = 3.7) from three vulnerable schools located in rural and urban areas of Guatemala were assessed. The results indicated that exposure to violence significantly moderates the effect of sociodemographic and socioeconomic variables in measures of depression, anxiety and health-related quality of life. Adolescents more exposed to violence reported higher levels of depression and anxiety, as well as lower levels of health-related quality of life. In contrast, food insecurity did not seem to influence psychological adjustment outcomes in this low-SES sample. These findings highlight the relevance of exposure to violence for mental health and well-being, and is a factor that should be considered when designing public health policies to promote children and adolescents’ welfare.

## 1. Introduction

Multidimensional poverty affects up to 1300 million people around the world, and usually entails living with lack of food, sub-optimal access to health services, education and daily life facilities. In turn, this impedes the attainment of adequate levels of well-being [1]. Children make up half of the people who suffer from multidimensional poverty, and one consequence for those children developing in such vulnerable conditions is that it places them in a disadvantaged position that tends to perpetuate poverty through successive generations [2]. Childhood poverty is a complex phenomenon which is not only related to income or poor nutrition, but also to many other factors that affect children’s well-being, such as lack of stimulation, overcrowded homes, lower parental education, or higher levels of parental stress or anxiety [3]. This condition is not exclusive of developing countries as one out of five children living in developed countries also experiences such vulnerable conditions [4]. The percentage of poor children living in urban areas is rapidly increasing, and nowadays one out of three urban children lives in marginal conditions. This often means living without secure housing, overcrowding and unsanitary conditions often accompanied with high unemployment, pollution, traffic, crime and exposure to violence, high cost of living, poor access to social services and competition for resources [5].

Childhood poverty is strongly related to children and adolescents’ family socioeconomic status (SES). This theoretical construct is a combined measure of economic and social status indicators that have been found to positively correlate with better health outcomes [6]. Although there are different positions regarding all SES indicators that should be considered, most studies agree with three common measures of family characteristics: family income per capita (sometimes referred to as income-to-need ratio), parents’ educational level and parents’ occupation. Family SES impacts children’s lives in a complex way, and many studies have shown how SES influences the adequate fulfilment of processes related to physical, emotional and cognitive development, standing out as an important factor related to their health and well-being [7,8,9,10,11].

Health is defined by the World Health Organization (WHO) as a state of complete physical, mental and social well-being and not merely the absence of disease or infirmity. Consequently, mental health is defined as a state of psychological well-being determined by biological, socioeconomic and environmental factors, and not only by the absence of mental disorders. This state includes neurological, psychological and social conditions that allow individuals not only to manage their thoughts, emotions, behaviors and social interactions, but also to find a place for themselves in their communities and enjoy life [12]. The effort that individuals make to manage their physical, psychological and social conditions results in their ability to adjust themselves to life needs. This ability is commonly stated as psychological adjustment, and depression and anxiety usually appear when individuals are unable to achieve a successful adjustment [13].

Neuropsychiatric disorders are estimated to represent about 14% of the global burden of disease worldwide, mainly related to the chronic nature of depression and other common mental disorders [14]. Despite such estimates highlighting the importance of mental disorders for public health, national health systems have not always paid enough attention to them. Thanks to current advances in neuroscience, today we know that physical and mental processes are interconnected, so it is important to promote the idea that there is no health without mental health. However, if this problem is still present in the adult population, it is easy to imagine how mental health issues have often been overlooked in children and adolescents, and this is especially true with children living in vulnerable conditions. The WHO has estimated that one out of five adolescents may suffer a mental disorder each year, with depression being one of the main conditions [15]. Fortunately, international initiatives such as the 2030 Agenda proposed by the United Nations through the Sustainable Developmental Goals are aimed at eradicating the differences that poverty causes around the world, and mental health is becoming a key component of this global agenda.

The latest data show that mental disorders are the main cause of disability among children and adolescents. Mean global prevalence data for mental disorders in minors between ages 5–17 was 6.7% (depression and anxiety showed 6.2% and 3.2% of coverage prevalence, respectively). However, the prevalence is unknown in most of the countries with up to 66% of countries having no data available for any childhood mental disorders [16]. This is especially the case in middle and low-income countries, with data generally unavailable in most of them. In the case of Guatemala, there is no public data related to the prevalence of mental disorders in children and adolescents, but a recent analysis of Guatemala City youth’s health showed that around 23% of them reported abnormal mood (extremely sad or happy, withdrawn, suicidal, anxious or aggressive) [17]. According to the Diagnostic and Statistical Manual of Mental Disorders, 5th edition [18], anxiety refers to a general state of worry that the individual finds hard to control, persists at least 6 months and, among other implications, has a negative impact on individual health outcomes. General anxiety is characterized by feeling fatigued, irritable as well as having muscular tension and presenting sleep problems. Furthermore, the DSM-V defines depression as a mood disorder characterized by feeling sadness, having feelings of hopelessness and experiencing loss of interest in previously preferred activities. Typically, major depressive symptomatology is generally related with having a depressive mood most of the day, diminished ability to focus on daily activities and recurrent negative thoughts, among other characteristics. Due to the nature of both disorders, anxiety and depression have been related in the literature [19], since both include negative mood behaviors and feelings as part of their general symptomatology. In this sense, health, psychological adjustment and quality of life are interconnected concepts, since quality of life has been used to define a compendium of subjective perceptions about psychical health, psychological state, personal beliefs and social well-being [20].

The impact of socioeconomic factors in children and adolescents’ mental health have been studied in several Latin American countries. For example, Cervantes et al. [21] studied a sample of children living in Colombian slums, finding that nearly half of them were at risk of suffering from anxiety. In a study conducted in Brazil with vulnerable adolescents results showed that participants had feelings of loneliness and insomnia [22], which are factors related to depressive mood. Moreover, in a study conducted in Peru, results showed that poverty has a negative impact in risk of suffering from familiar mental health problems, as well as malnutrition [23].

In high-income countries like the US, longitudinal and prospective studies have found evidence that childhood poverty predicts adult chronic physiological stress and altered emotional regulation neurocircuitry that are linked to higher externalizing and internalizing symptoms (e.g., anxiety and depression), cognitive deficits, helplessness behaviors and reduced psychological well-being [24,25]. However, without measures from middle and low-income countries it is almost impossible to form specific diagnostics at national or regional levels, so increasing available data on mental health among children and adolescents, especially in low-SES backgrounds, is urgently needed.

Evidence from international studies about psychological adjustment in children and adolescents highlights that, in terms of age, it seems that depressive and anxious symptoms are considerably more common in adolescents than in children [26]. This tendency increases with age and is probably due to the fact that as adolescents are in a developmental state in which they are more aware about what is happening in their environment [27]. The depressive symptomatology experienced by female adolescents has been a cause of concern in studies in different countries, with females assessed as having higher levels of depression compared with males of a similar age [28,29,30,31]. Nevertheless, when authors of a meta-analysis examined data regarding how the incidence of depression was distributed in adolescents from low-income settings, although they confirmed the high incidence of depression among adolescents, the influence of gender was not present once socioeconomic indicators were taken into account [32]. There is evidence of higher depression scores in urban adolescents in low-SES settings in comparison with rural groups [33], but when different rural and urban areas are compared, these differences in the depressive symptomatology reported by adolescents may disappear. Regarding anxiety disorders, and as for depressive symptoms, international evidence shows that these externalizing behaviours tend to be more common in female adolescents from low-SES urban settings, compared to males [34,35]. Finally, in terms of quality of life and psychological well-being, available data point to an inverse pattern compared to depression and anxiety symptomatology. In this case, younger male children from high-SES backgrounds tend to perceive themselves with better quality of life and psychological well-being compared to other gender, age and SES groups [8,29].

Guatemala is a diverse, multilingual and multicultural country, with an estimated population of more than 15 million inhabitants. Around 41% of the population is indigenous, with four peoples (Maya, Garífuna, Xinka and Ladino) who represent unquestionable cultural wealth, but also face marked conditions of inequality. Although most of the Guatemalan population is rural, 40% of its inhabitants already live in cities. It is estimated that 50% of the total national population is under 18 years of age, which makes it a young country with a population growth rate of 2.5% and a literacy rate of 55.6% [36]. Guatemala remains one of the poorest countries on the American continent, with high rates of inequality and violence, in which structural problems persist such as racial discrimination, social inequality, a profound situation of poverty and exclusion, an unstable political situation and an aberrant lack of access to justice, constituting an obstacle to the full enjoyment of human rights.

According to data from *Fe y Alegría Guatemala* Educative Foundation [37], the most disadvantaged rural regions of Guatemala have poverty rates of 94.5% and extreme poverty of 55.4%. Almost 100% of the rural population is of indigenous origin and their main source of income is agriculture. Primary education coverage is close to 100%, but its completion rate is 15%, and secondary schools low overall rates of enrollment. The gender inequality surrounding access to education is notorious: in more advanced levels of secondary and high school, ratios of 2 women for every 100 men can be reached. This may be related to another cause of concern in Guatemala: early pregnancy, defined as the number of pregnancies in females under 20 years of age [38]. Of the total number of early pregnancies in the country, 80% involved females under 15 years of age, and there is an average annual death rate of 175 women due to the lack of prenatal care and malnutrition. Moreover, the level of malnutrition in the general population is alarming: 65%. Only 28.7% of the homes have access to drinking water and basic sanitation. Therefore, in rural areas, the population still works the land in subsistence conditions, with a high degree of food insecurity and little access to education (mainly by decision of families who, for economic or cultural reasons, only send some of their children to school, with girls usually being the ones who first drop out when families cannot cover the expenses). Evidence from international studies clearly reflects that food insecurity has been significatively associated with physical and mental health measures, with sociodemographic and socioeconomic factors being important related factors [39]. For instance, in a study conducted with a sample of US adolescents, food insecurity during the previous year was associated with mood and anxiety disorders, among others [40]. Another study compared US children and adolescents’ mental disorders as an outcome measure, considering sociodemographic and socioeconomic factors, with results also showing that children from households in which food insecurity was higher, the probability of experiencing mental disorders increased (e.g., anxiety, mood, or behavioral disorders) [41].

The suburban areas that are on the outskirts of Guatemalan cities are characterized by being communities with significant problems in basic services. These are very densely populated areas, where informal employment (informal street and market sales) is the main source of income for most households. A large percentage of these families are single parents, with women as heads of households, and therefore responsible on many occasions for extended families that survive on a single salary in this type of underground economy. Parents’ educational level is not high, and few have reached technical or higher education. Although most houses have access to some basic services, normally many people live together in confined spaces, which can generate adverse situations for study or recreation. Although the basic diet is usually covered, sometimes people living in these areas often do not consume three meals a day. Additionally, it is likely that the possibilities of recreation for children and adolescents in these contexts is not high, and access to cultural offerings at home, such as books, is limited. One of the main problems affecting these suburban areas is the elevated presence of gangs and other violent groups in the neighborhoods. Violence rates have increased significantly in recent years, and assaults, shootings, house robberies, extortion and murders occur frequently. The youth living in these suburbs not only suffer the disadvantages of living in socio-economically disadvantaged areas, but this is compounded by a high exposure to violence and the risk of becoming part of gangs. Consequently, the role of schools, and of working with families, is key to avoid children becoming linked to gangs [37]. Exposure to violence used to be positively related to both depression and anxiety, and negatively to health-related quality of life [42]. The existence of a relationship among poverty, violence and mental health has been studied in several Latin America and Caribbean countries. For instance, Samms-Vaughan & Lambert [43] studied the implication of poly-victimization in Jamaican adolescents’ mental health, finding that the associated factors: poverty and violence, not only have negative consequences in adolescents’ mental health, but also they involve consequences in terms of intellectual functioning and behavioral outcomes. In this regard, there are remarkable studies conducted in especially vulnerable populations in Colombia and in the border region of the United States and Mexico. Gómez-Restrepo et al. [44], studied a sample of displaced children as a consequence of army conflict in Colombia. According to the reported data, these children were more likely to suffer from symptomatology related to anxiety and post-traumatic disorders. Moreover, in the case of children that live along the American-Mexican border [45], results showed that those children who were exposed to poverty and collective violence reported more problems in psychosocial, as well as in mental health indicators, in comparison to those who experienced poverty without collective violence.

Studies aiming to assess the implications of living in violent urban settings have shown that girls’ mental health is commonly affected by the experience of victimization [46]. Moreover, in a study conducted with a large sample of urban Chinese adolescents [47], results suggested that victimized adolescents were likely to have difficulties in some mental health outcomes, such as depressive symptomatology. Furthermore, in a study conducted in Vietnam, relations between different familial characteristics, types of violence as emotional maltreatment and depression were found in adolescents [48]. Nevertheless, the impacts in mental health outcomes caused by exposure to violence have also been observed in rural communities, showing similar patterns [49]. Research about the relation between self-perceived quality of life and exposure to different types of violence (e.g., maltreatment) by children and adolescents is limited. Nonetheless, some authors from developing and developed countries have focused on this topic. In developing nations, and especially in Asia, studies that have tried to connect diverse types of exposure to violence with mental health indicators, as well as Health Related Quality of Life, have been conducted. The results showed that peer-victimization [50] and different types of poly-victimization [51], end up affecting quality of life scores in adolescents. Furthermore, the relation between violence exposure and scores in quality of life have been studied in developed countries. For example, in a study conducted in Germany, a negative relation between both variables in the case of maltreated adolescents was found [52]. In the same country, Schlack et al. [53], obtained similar results when they compared victimized and non-victimized children from different SES backgrounds in terms of emotional problems such as feeling worried, unhappy and fearful, since victimized children tended to have greater mental health disturbances. Some studies have analyzed different components of quality of life (e.g., physical, psychological or social), finding that children obtained higher scores than adolescents in the overall score, male adolescents usually scored higher than girls in every component and that participants coming from slums scored lower than those who came from another types of neighbourhoods [54,55]. Congruently with these findings, hope and exposure to community violence have been found as statistically significant predictors of urban adolescents’ well-being [56].

## 2. Aim of Study

The main aim of the present study was to analyze the relations that sociodemographic variables (gender, age, and rural/urban habitat) and socioeconomic factors (parents’ educational level and family income per capita) have with low-SES Guatemalan children and adolescents’ psychological adjustment (depressive and anxious symptoms, and self-perceived health-related quality of life). Additionally, we aimed to analyze how these relations could be mediated by food insecurity and violence exposure, two of the main problems that these children and adolescents face. Authors’ main hypothesis was that sociodemographic (gender, age and habitat) and socioeconomic (educational level and income per capita) variables would influence vulnerable children and adolescents’ psychological adjustment, being this relation moderated by exposure to violence and food insecurity. The specific hypotheses were (a) female adolescents from urban settings will score worse in psychological adjustment outcomes; (b) children whose parents have higher income and educational level will have better psychological adjustment outcomes; and (c) violence exposure and food insecurity will moderate the relation between sociodemographic and socioeconomic variables, worse scores in these variables being related with worse scores in psychological adjustment outcomes.

## 3. Materials and Methods

### 3.1. Participants

A convenience selection was implemented in order to obtain a balanced sample in age and gender, in each educative center. Complete classrooms of each course were invited to participate in the study. Then, a random selection was applied among authorized participants to balance age and gender groups. The study sample included 185 participants from which 94 were girls (50.8%) and 91 boys (49.2%) ranging from 6 to 17 years of age (M = 11.82, SD = 3.7). The total sample was divided into two groups: children and adolescents, being taken as adolescents those participants whose age was 12 or over. The children group included 89 participants (M = 8.39, SD = 1.9) from which 43 were girls (48.3%). A total of 96 participants (M = 14.99, SD = 1.5) conformed the adolescents’ group 51 of them being girls (53.1%). They belonged to 2 rural and 1 urban educative centers of Fe y Alegria organization in Guatemala, being those schools were chosen to take part in the study due to the low-SES and especial vulnerability of their students.

The urban educational center was in Villanueva district (from now VIL), in the suburbs of Ciudad de Guatemala. The location of this school placed the children in a vulnerable position because of the violence they were surrounded by, since the school was placed in a confluent point between the influence areas of two criminal gangs. Both rural educational centers were in Totonicapán department, and both were characterized by the scarcity of resources suffered by their students. The school placed in Santa Lucía La Reforma (REF) was a small center with a multigroup system in which children of different ages shared the same classrooms across primary level. A large proportion of their students were Kiche’ indigenous population, and therefore this school had an important number of bilingual students proficient in Spanish and K’iche´ languages. The second rural school was an educational center placed in Santa María Chiquimula (CHI). It was bigger and had many students from different ranks of age, including adolescents in secondary level and bachelor. This educational center also has a considerable number of K´iche´ indigenous students. Because of the difficulties that some students faced in going to school, this educational center offered morning and evening classes.

All participants met the following inclusion criteria: (a) being between 6 and 17 years of age, (b) being enrolled in low-SES and especially vulnerable educative centers, (c) being Spanish-language speakers, (d) lack of personal history of nervous system disease that could case neuropsychological shortfall (e.g., epilepsy, multiple sclerosis…) and (e) lack of personal history of psychiatric disorders (e.g., bipolar disorder, psychosis…).

### 3.2. Instruments

Participants’ caregivers answered a sociodemographic questionnaire that included SES indicators such as family income, number of inhabitants at home, or years of education. Caregivers also completed a clinical questionnaire that included information about the child health status during the pregnancy, delivery, diagnosis during infancy (e.g., neurodevelopmental or psychiatric diagnosis), as well as any other kind of health difficulties (e.g., visual or motor problems), and information about the current child medication intake. From the total, only eleven respondents (5.9%) were not parents, most of them being grandmothers, aunts or older sisters. The sample selection and characterization data were obtained from these questionnaires, which were similar to those used by Rivera & Arango-Lasprilla [57].

The violence exposure scale revised (VEX-R) [58] is a tool to explore the frequency the child has received or witnessed violent acts. The VEX-R represents through the cartoon characters José and María (in the Spanish version) a set of 22 pictures of directly received or witnessed violent acts with increasing severity. The child had to indicate how often he or she had experienced the same events, a punctuation being obtained in each item: never (0), one time (1), some time (2) or many times (3). The score range of the scale is from 0 to 66. Even though there is limited psychometric data available yet, this tool has been successfully used in samples with low-SES settings [59]. This scale presented good reliability in the present study, Cronbach’s α = 0.87, Guttman’s split-half reliability = 0.77.

The Latin-American and Caribbean Latin-American food security scale (ELCSA) [60] was validated in the Guatemalan population after its application in a study by the Food and Agriculture Organization of the United Nations in 2011, with good psychometric properties. In that study, its measurement was proven to be correlated with sociodemographic variables related to poverty. This scale was answered by the caregivers and its content had two parts. In the first section, caregivers were asked about how safe they feel in relation to the food they can provide to the family. The assessment of this part offered a score between 1 to 15 in which higher scores reflect higher food insecurity. In the second section, it was reported how many days per week the family consumes a certain type of food [e.g., vegetables, meat and fish, sugary foods). In the present work, this scale showed good reliability, Cronbach’s α = 0.85, Guttman’s split-half reliability = 0.80.

### 3.3. Outcome Measures

The Kidscreen-10 questionnaire, created by the Kidscreen group [61], assesses through ten questions how the child or adolescent self-perceives his or her quality of life in regard to their own health and well-being. This questionnaire is a shorter version of Kidscreen-52 scale, which has been previously used to assess quality of life related with health in Latin-American adolescents [62]. The reliability and validity of this questionnaire has been successfully assessed [63]. Some of the items included in this scale were: *Have you felt good and fit?,* or *Have you been able to do the things that you want to do in your free time?*. Each of the 10 items was measured in a Likert scale from 0 to 5. Higher scores indicate higher levels of well-being and quality of life. In the present study, this instrument presented good reliability, Cronbach’s α = 0.76, Guttman’s split-half reliability = 0.81.

The anxiety subscale of the Revised Child Anxiety and Depression Scale (RCADS-subscale) [64] was used in children of 3th course of primary education and over. The RCADS has shown good psychometric properties, as well as adequate internal consistency across different countries, contexts and languages [65]. In this study, the participants only had to answer to the items included in the generalized anxiety factor [66]. This 6-item subscale had a Likert-type format in which the participant had to say from 0 to 3 how often he/she have felt like the item indicates (never, sometimes, often and always). The score range is from 0 to 18. Raw scores can be transformed to standardized scores depending on the child grade. Standardized scores ≥65 are a cause of concern. Good reliability scores were observed in the present work, Cronbach’s α = 0.74, Guttman’s split-half reliability = 0.75.

The self-reported Children´s Depression Inventory (CDI) [67] is appropriate to be used in children and adolescents. The psychometrics index to the Spanish adaptation has been adequate in both internal and external validity [68]. This questionnaire has 27 items which assess aspects as negative humor, ineffectiveness, low self-esteem, social withdrawal and pessimism through two subscales; dysphoria and negative self-esteem. In each item, there are three possible responses that score 0, 1 or 2, being higher punctuations related with higher probability of having depressive symptomatology. The cut-off score is 19, higher scores being considered a cause of concern. The score range is from 0 to 54. In the present study, it showed good reliability, Cronbach’s α = 0.83, Guttman’s split-half reliability = 0.82.

### 3.4. Procedure

This study complies with the provisions of the Declaration of Helsinki and was approved by the Andalusian Biomedical Research Ethics Committee. The assessment was conducted by three local trained psychologists. The psychologists and directors of each educational center met with the parents aiming to explain the evaluation process and asking them for permission for their children’s participation. In order to allow the students enrollment, parents were asked to sign the informed consent and to complete the sociodemographic and clinical questionnaires. Once the final sample was selected, psychologists individually assessed children in comfortable and well-illuminated rooms provided by the educative centers. The assessment took around 25 min for each participant.

### 3.5. Data Analysis Design

First, descriptive statistics (e.g., mean and standard deviation) were examined for food insecurity, exposure to violence, depression, anxiety and quality of life, also performing bootstrap analyses (95% Confidence Interval). Kolmogorov–Smirnov test (with Lilliefors correction) for normality was conducted. Results indicated that all study variables did not follow a normal distribution (depression: KS = 0.14, *p* < 0.001; quality of life: KS = 0.12, *p* = 0.001; anxiety: KS = 0.09, *p* = 0.043; exposure to violence: KS = 0.09, *p* = 0.023; food insecurity: KS = 0.18, *p* < 0.001), so that non-parametric analyses were carried out.

Second, Spearman bivariate correlations were performed to examine the associations between these variables. Third, demographic effects on study variables were analyzed by calculating differences by gender (boys vs. girls), age (children vs. adolescents) and habitat (rural vs. urban), with separate Mann-Whitney U-tests. The associations with socioeconomic variables (e.g., mean in parental years of education and income per capita) were analyzed with bivariate Spearman correlations.

Fourth, three separate hierarchical regression analyses were carried out to examine the joint effects by demographic and socioeconomic variables, food insecurity and exposure to violence, on psychological adjustment variables; that is, depression, anxiety and quality of life. Fifth, based on previous analyses, regression-based moderation analyses were conducted by examining the effects by the interactions between food insecurity and/or exposure to violence x every demographic and socioeconomic indicator, on psychological adjustment variables. Bootstrap analyses were performed for calculating confidence intervals of the coefficients in these regression analyses. Also, collinearity tests were conducted. These statistiscal analyses were carried out with the program SPSS 21.0 [69].

## 4. Results

### 4.1. Descriptive Statistics and Bivariate Correlations

Table 1 and Table 2 presents descriptive statistics of study variables and bivariate correlations among them. Reduced means were observed in food insecurity and depressive symptoms, while moderate ones were found in exposure to violence and anxiety. Notable scores were detected in quality of life. Concerning the associations between the variables, results showed that exposure to violence was positively related to both depression and anxiety, and negatively to quality of life. No significant associations were found regarding food insecurity. Moreover, quality of life was negatively associated with depression and anxiety, while these two variables were positively interrelated. Negative associations were found between food insecurity and SES variables (both educational level and income per capita). Positive association was found between depression and income per capita. Educational level and income per capita were positively interrelated.

### 4.2. Demographic and Socioeconomic Effects on Study Variables

Table 3 shows effects by demographics (e.g., gender, age and habitat) and socioeconomic variables (e.g., educational level and income per capita) on food insecurity, exposure to violence, depression, anxiety and quality of life. Gender differences were observed in depression, with girls (M = 10.49, SD = 7.44) presenting higher means than boys (7.63, SD = 5.55), and in quality of life, with higher scores in boys (M = 39.45, SD = 5.42) than girls (M = 36.93, SD = 7.19). Age differences were observed in depression, anxiety and quality of life. Adolescents presented more depressive symptoms (M = 11.97, SD = 7.54) than children (M = 5.97, SD = 3.76), as well as more anxious symptoms (adolescents: M = 9.06, SD = 3.68; children: M = 6.61, SD = 2.95). Children presented more quality of life (M = 40.90, SD = 4.70) than adolescents (M = 35.64, SD = 6.90). Concerning habitat, differences in exposure to violence and depression were found. Participants from urban habitat reported more exposure to violence (M = 25.60, SD = 11.08) and depression (M = 10.54, SD = 6.54) than those from rural habitat (M = 16.76, SD = 8.73, and M = 8.23, SD = 6.70, respectively). Food insecurity showed no differences across gender, age or habitat. Finally, concerning socioeconomic variables, results indicated that greater parental educational level and more income per capita were associated with lower food insecurity.

### 4.3. Hierarchical Regression Analyses and Moderations by Demographic and Socioeconomic Variables

Table 4, Table 5 and Table 6 present the results of three hierarchical regression analyses to examine the respective joint effects on depression, anxiety and quality of life, by demographics, socioeconomic variables, food insecurity and exposure to violence. In the three criteria variables, effects by age and exposure to violence were significant, so that more depression and anxiety, and less quality of life, were observed in adolescent participants with more exposure to violence. Explained variable above 20% was observed in both depression and quality of life.

Because only exposure to violence showed significant effects on psychological adjustment variables, moderation analyses were conducted to examine how those effects by exposure to violence could vary based on levels in demographics (gender: boys, girls; age: children, adolescents 12 years old or more; habitat: rural, urban) and socioeconomic variables (50% low vs. 50% high). Table 7, Table 8 and Table 9 shows results from moderation analyses.

**Figure 1:**
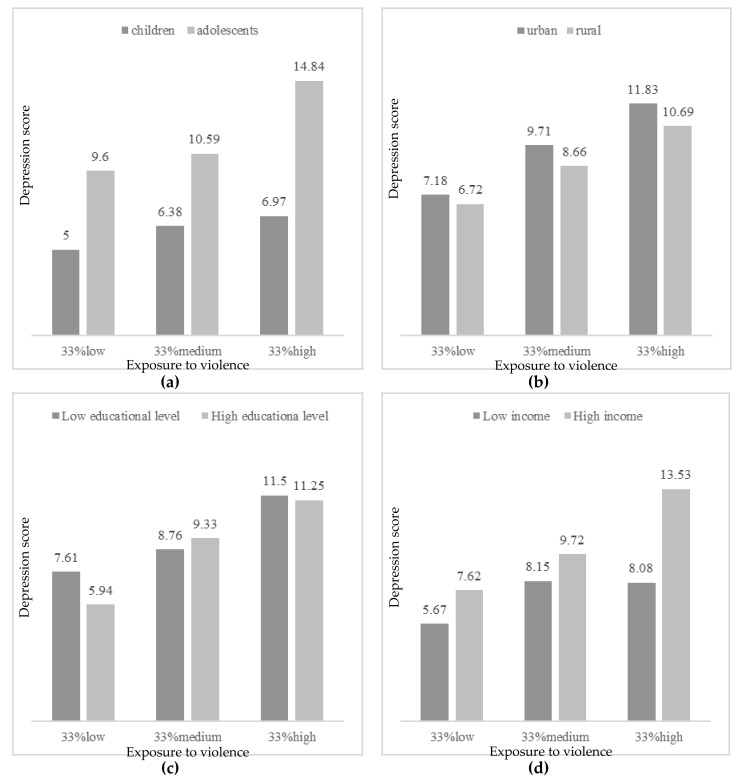
Depressive symptoms by exposure to violence (33% low, 33% medium, 33% high) according to (**a**) age (children vs. adolescents); (**b**) habitat (urban vs. rural); (**c**) educational level (low vs. high); and (**d**) income per capita (low vs. high).

**Figure 2:**
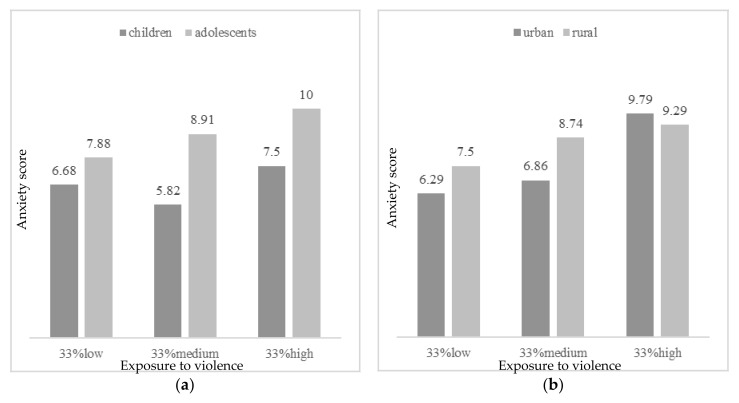
Anxious symptoms by exposure to violence (33% low, 33% medium, 33% high) according to (**a**) age (children vs. adolescents); and (**b**) habitat (urban vs. rural).

**Figure 3:**
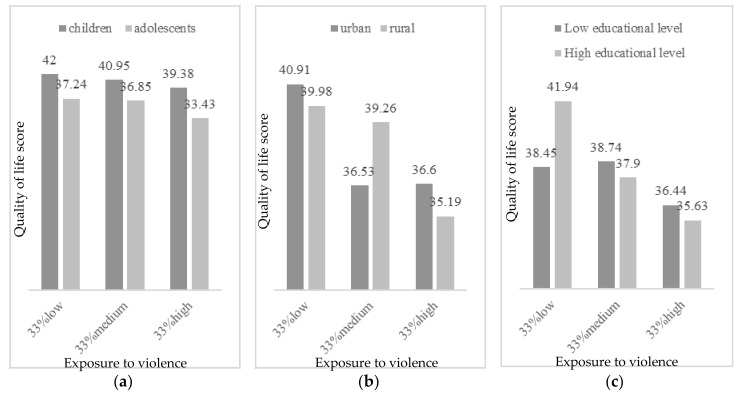
Quality of life by exposure to violence (33% low, 33% medium, 33% high) according to (**a**) age (children vs. adolescents); (**b**) habitat (urban vs. rural); and (**c**) educational level (low vs. high).

Concerning depression, the effect by exposure to violence was moderated by age, habitat, educational level and income per capita. As Figure 1 presents, more depressive symptoms were observed with high exposure to violence in adolescents, from urban habitat, and more income per capita (more than 200 Q a month). In participants with parents with low educational level (less than 6 years of education), more depressive symptoms were detected even in low exposure to violence. Concerning anxiety, age and habitat significantly moderated the effect by exposure to violence. Thus, more anxious symptoms were found in participants with more exposure to violence, in adolescent years and from urban context (Figure 2). However, in low or moderate levels of exposure to violence, more anxiety was reported by participants from rural areas. Finally, regarding the effect on quality of life by exposure to violence, it was found to be moderated by age, habitat, and educational level. Figure 3 describes the levels of quality of life by exposure to violence (low, moderate or high), age, habitat and educational level. Greater quality of life was reported by children with low exposure to violence, from urban habitat and parents with high educational level (more than 6 years of education). In cases with moderate levels of exposure to violence, more quality of life was reported by those from a rural habitat.

## 5. Discussion

The objective of this study was to analyse the implications that sociodemographic and socioeconomic factors have in low-SES Guatemalan children and adolescents’ mental health and well-being, and how these relations were mediated by the experiences of food insecurity and the exposure to violence. Therefore, the authors’ main hypothesis was that sociodemographic (gender, age and habitat) and socioeconomic (educational level and income per capita) variables would influence vulnerable children and adolescents’ psychological adjustment (depression, anxiety and health-related quality of life), being that this relation was moderated by violence exposure and food insecurity.

Bivariate correlations showed that the violence exposure was, like in other studies, positively related to both depression and anxiety [19], and negatively to health-related quality of life (HRQoL) [42]. Despite the relative lack of studies focused on children’s mental health in developing countries, the presented results agree with the existing literature. The results related with mental health are congruent with studies conducted in the Brazilian adolescent population exposed to violence in which female adolescents reported higher levels of anxious and depressive symptomatology such as sleep disturbances and having feelings of loneliness [22]. Negative effects on mental health outcomes experienced by children in Jamaica have also been shown in regard with violence exposure [43], being this finding congruent with the presented results.

As expected, depression and anxiety were negatively correlated with HRQoL. However, contrary to hypothesis [40], no relations with mental health factors were found in regarding food insecurity. The effects of demographics and socioeconomic factor on study variables agree with the previous evidence reported. Factors related with depressive and anxious symptomatology are similar in the general population, and the results of our sample were expected to be in the same direction due to the usual comorbidity between both of them [70]. Regarding depression scores, the obtained results agree with the literature in terms of gender [28,29,30,31] and age differences [26,27]. In this case, female adolescents obtained higher scores in depressive symptomatology. The obtained results are also congruent with previous literature in terms of anxiety, which tend to be more common in female adolescents like depressive symptoms [34,35,71]. Regarding habitat (rural vs. urban), the results showed that the urban group experienced higher levels of depression than the rural group, supporting the evidence previously found in low-SES settings [33]. The fact of living in crowded and stressful areas, with deficiencies in the health services and education access, could be a key factor to understand how depression scores and urban contexts are connected [5]. Moreover, the higher community violence experienced by the urban cohort of our study seems to be an important point to understand the results related with mental health outcomes. Living in suburban settings and violence exposure is commonly related in different low-SES contexts, and both conditions usually affect adolescents’ mental health [72]. Taking previous literature into account, it would make sense to think that sociodemographic and socioeconomic variables related to self-perceived HRQoL would be the opposite of those described for the variables of depression and anxiety, and our results were congruent with this scenario, showing female adolescents had the lower HRQoL scores [54,55]. In order to interpret the obtained results in this variable, it is necessary to consider that according to the literature, younger children tend to overestimate the way in which they perceive the satisfaction with their lives, being that this estimation is moderated in older ages [8]. It is presumable that the higher HRQoL’s index is reported by the youngest, since at early ages, children may not be aware of the difficulties there are surrounded by due to this awareness gradually developing during the adolescent period. In any case, both children and adolescents tend to show an optimistic bias compared to adults from the same SES when self-assessing their quality of life and well-being [8].

Hierarchical regression analysis showed that only age and exposure to violence significantly explained the variance in the psychological adjustment variables considered in this study; that is, adolescent participants who were more exposed to violence obtained higher scores in depression and anxiety and lower scores in HRQoL. Age and violence exposure are common factors related with the three psychological adjustment variables assessed [19,42,73]. However, neither food insecurity nor gender, habitat or socioeconomic variables significantly explained the changes observed in these psychological adjustment variables. In the moderation analysis regarding violence exposure and depression scores, the effect of violence to depression levels was higher in adolescents who lived in the urban area, in households with less educational level and more income per capita. These results agree with the researchers’ hypothesis, since the assessed urban cohort is more exposed to community violence due to the context they are surrounded by (e.g., the school is located in a confluent point between two criminal gangs or “maras”), what may interfere in adolescents’ psychological adjustment, as they are much more exposed than the children to the influence of these gangs and have more awareness of this situation. These results agree with others studies that have showed the implication that different types of violence exposure has for urban adolescents´ mental health in depression, increasing these effects over time [74,75]. These effects have been shown as well in urban children who are exposed to violence [47,48]. Other studies conducted in Latin American and Caribbean countries where contexts of vulnerability are similar to the assessed sample in terms of poverty and violence, agree with the obtained results. Evidence that connect violence and poverty with decreasing children and adolescents’ mental health have been found in Colombia [44], Brazil [22] and Jamaica [43], as well as in the border between United States and Mexico [45]. All of these studies have added evidence to the field of the negative consequences that younger population may suffer due to the lack of resources and the social determinants associated to it. Similar results in terms of depressive symptomatology have also been shown in vulnerable adolescents from Asiatic countries such as China [47] and Vietnam [48]. Although this population corresponds to countries which have a different culture from the assessed sample, it supports the evidence that violence seems to negatively affect adolescents’ mental health all over the world.

Gender has been usually found to be a significant factor in this relation, being urban female adolescents are more affected in terms of depression and anxiety symptoms by violence exposure [76]. Although gender differences were observed in depression, with girls presenting higher scores than boys, this effect disappeared when joint effects by other demographics and socioeconomic variables were examined, as Kempfer et al. found in their meta-analysis [32]. Taking into account the analyzed sample, in which male adolescents can be involved in violence acts, as well as in violent gangs in a higher proportion than girls, some variables could interact being this the reason why there is a change in the relation between variables when other factors are included in the analysis.

Regarding parent’s educational level, higher scores of depression were found among participants from low educational level families when exposed to low levels of violence. However, this difference tended to disappear with medium and high levels of violence exposure. These results are partially congruent with previous literature which highlight parental education as a protective factor for internalizing behaviours problems like depression [7]. The effects of this protective factor could be insufficient for moderate and high levels of violence exposure, as most of our sample was in a low-education range when compared to the general population (the cut-off for low parental education was less than 6 years of education). Family income per capita also moderated the relation between violence exposure and depressive symptomatology in children and adolescents, but in this case, results were contrary to our hypothesis, as participants with high family income showed higher depression scores, especially when faced with high levels of violence exposure. In this regard, it is important to consider that the cut-off to distinguish the level of income in this study has been 200 quetzals (around 26$) per month for each member of the family, but most of the sample would be in a low-SES range compared to the general population [46,76,77]. A possible explanation for these results could be related with the complexity of these contexts, in which interaction effects among variables (income, urban context, violence, or depression) could be happening. In this regard, it would be important to consider that families with higher incomes from the poor population, could be afraid of losing their ownerships when living in dangerous neighborhoods, so this could explain the growing trend in the depression scores. Moreover, this could be related with the differences in the highest levels of violence exposure, in which the difference between familiar income are remarkable.

Regarding anxiety scores, authors expected results in the same direction than obtained in depression scores. The results were congruent with previous literature, in which has been shown that adolescents who have grown up in violent urban poverty settings have higher anxious symptomatology and distress symptoms [47,48,78,79,80], being this relation among variables also studied in a systematic review [75]. For instance, in a research in which a sample of African-American and Latino adolescents from low-SES backgrounds in US was studied, results showed that community violence exposure, favored the appearance of anxiety and depression after 12 months of the baseline assessment [81]. Such is the level of concern about the issue, that intervention programs have already been implemented in order to mitigate the negative impact that living in violent urban areas have on adolescents’ mental health outcomes [82]. Nevertheless, as was shown in Figure 2b, we have also found that when participants from rural areas are exposed to low and medium levels of violence, they tend to show higher levels of anxiety than their urban peers (and similar levels of anxiety when exposed to high levels of violence). Thus, children and especially adolescents from rural areas should be also be of concern when developing intervention programs to improve mental health of minors living in violent areas.

In relation to the effect on HRQoL by violence exposure; age, habitat and parental educational level were relevant factors to moderate the relation between variables. A higher level of quality of life was reported in children less exposed to violence from the urban area and whose parents were more educated. According to age, these results are congruent with other results since higher scores in depression and anxiety were obtained by adolescents, and both variables were negatively correlated with HRQoL. In terms of area, the moderation effects are difficult to interpret. Urban participants showed higher scores in HRQoL than rural participants when exposed to low or high levels of violence, but this pattern changed in medium levels of violence exposure, where urban participants showed lower scores in HRQoL (like the scores obtained in a high level of violence exposure). Children from urban areas generally experience more violent acts, and they may not integrate this information in such a negative way that it can influence their HRQoL self-perception. Only in cases of moderate levels of violence exposure children from rural areas had higher scores in HRQoL than urban participants, as hypothesized, but it is not easy to say why it happened only at this medium level. Maybe, the protective factors of HRQoL associated to rural settings, combined with the habituation to violence exposure in the urban one, could explain why rural participants maintained similar levels of HRQoL through low and medium levels of violence exposure, but this protection could be insufficient when they faced higher levels of violence. On the other hand, as a result of a higher exposure to violence of urban participants, the highest scores of HRQoL could be found among urban participants that faced low levels of violence (they may perceive themselves like quite fortunate compared to other urban peers), but once the exposure to violence increases they rapidly concern about their HRQoL, showing similar scores in medium and high levels of exposure (but not increasing the impact of violence over HRQoL in the high level probably due to their habituation to it). Regarding parents’ educational level, participants whose parents had higher educational level reported higher scores in HRQoL when they were exposed to low levels of violence. However, once the exposure to violence increased, this difference almost disappeared and even participants from low educational level families showed slightly better scores in HRQoL. These results may be showing that the protective factor of HRQoL associated with parental educational level would be only effective in low violence exposure settings. These findings are congruent with previous studies that found a higher impact of violence exposure over adolescents’ HRQoL [42,54], and partially congruent with studies that found better HRQoL and well-being among participants from rural areas (or less violent settings) and belonging to higher levels of parental education families [8,43]. It is difficult to connect these results with previous literature since little research has been conducted connecting violence exposure and subjective well-being scores in children and adolescents. These results are congruent with studies conducted in developing countries such as in China [50] and Vietnam [51]. In this last country, higher levels of victimization and violence exposure were related with decreased scores in HRQoL in a sample of high-school participants [51]. The obtained results are also congruent with those found in developed nations such as Germany, where the exposition to multiple types of violence have shown a negative relation with HRQoL scores in adolescents [52,53].

Contrary to the authors’ hypothesis, food insecurity was not considered a significant mediation variable between the sample’s sociodemographic and socioeconomic characteristics and psychological adjustment indicators, as had been found in previous studies [30,31,32]. The manifestations of low-SES are diverse, and the lack of food is just one of them. It was expected that food insecurity would mediate between socioeconomic and sociodemographic variables and outcome variables. The fact that parents experienced worries about their possibilities of providing enough food to the family, was expected to interfere in children and adolescents’ mental well-being. A possible explanation to the obtained results could be related with the different perception that children and parents could have in relation with food quantity and quality. Maybe children and adolescents do not perceive the lack of resources in the same way that their parents do, being other described factors of our sample more implied in their psychological adjustment. Another explanation for the absence of effect of food insecurity over children and adolescents mental health and HRQoL could be related to the sample characteristics, as previous studies tend to use bigger national samples and more sociodemographic and socioeconomically diverse than our low-SES Guatemalan sample, where the effects of food insecurity over psychological adjustment could be shadowed by the lack of participants of higher SES backgrounds.

Considering all previously described implications that variables like violence exposure may have for adolescents’ mental health and well-being, the authors of this study consider that it is necessary to promote and facilitate the growth and development under equal conditions for all children and adolescents. In regarding the practical applications that these results may have, they would help to people who work with these vulnerable minors to go deeper in these factors that are being revealed as important features to help them to reach a better psychological adjustment. Furthermore, the obtained results should be considered when designing politic strategies to ensure the protection of children and adolescents’ welfare from low-SES backgrounds of Guatemala. This study may help to better understand the implications that violence exposure has for children and adolescents who live in vulnerable conditions. Moreover, the obtained results showed that food insecurity experienced by parents, does not seem to moderate mental health outcomes in these children and adolescents, but it does not mean that food insecurity is not an important factor in bigger pictures. As has been shown, violence exposure seems to be the most significant factor that should be considered when designing intervention programs to promote vulnerable children and adolescent’s mental health and well-being.

### Limitations and Future Directions

It is important to consider that some questionnaires such as the food insecurity one were self-reported by parents, and therefore they are subject to social desirability. This fact could affect to the underestimation of reported food insecurity. In order to avoid this possible bias, could be interesting to implement the inclusion of multiple informants as well as the use of objective measures in future studies. Furthermore, in relation to mental health questionnaires, it would be interesting to use suitable scales, adapted to Guatemalan children and adolescent’s population (e.g., adapting questionnaires to native languages -most of them only used orally- with which some participants could feel more comfortable answering health and emotional issues). Other limitations come from the number of participants, which may directly affect external validity. Also, only participants from low SES backgrounds were included in the study. Thus, increasing the sample size and including higher SES backgrounds would improve the strength of the findings in further researches.

On the other hand, the scarcity of studies that consider health-related quality of life reported by children and adolescents living in vulnerable conditions is notable. Therefore, it would be interesting to investigate in depth in future studies the implications that this variable may have in these children that grow up in such vulnerable conditions. Moreover, authors of this study suggest the application of longitudinal studies in order to better know how relation among variables’ directionality is along the time. If the temporality is significant, it could help to stablish suitable intervention programs depending on the developmental stage the children are in.

Otherwise, after analyzing the consequences that living in vulnerable settings has for children and adolescents, it would be necessaire to control any other variables that may have an impact how stress affects these children. Interesting variables to include in future analysis would be the parents-child relationships or children and adolescents’ coping styles, for instance. Finally, after analyzing the effect that violence exposure has for children and adolescents’ psychological adjustment, it would be interesting to go further in future researches in terms of type of violence exposure. There are studies that have found that to witness and to be a victim of a violent act have different implications for young population [83], meanwhile other studies reported the opposite [84]. It would be also interesting to study the implication that violence exposure has in adolescents’ mental health and well-being, in regarding to the person who receive the violent act. These results may help to better understand how some variables can moderate the negative impact that violence exposure have in children and adolescents, being this information of great importance when creating intervention programs.

## 6. Conclusions

- Violence exposure and age had significant effects on psychological adjustment. Adolescents more exposed to violence obtained higher scores in depression and anxiety, and lower scores in health-related quality of life.
- Higher depression scores were found in adolescents from urban settings, with lower parents’ educational level, and higher family incomes.
- Higher anxiety scores were found in adolescents from urban settings, who were more exposed to violence, but adolescents from rural settings should be also under concern.
- Higher levels of health-related quality of life and well-being were found in children less exposed to violence, from urban settings, and whose parents had higher educational level.
- Food insecurity had no significant effect on the variation of psychological adjustment in this sample of low-SES Guatemalan children and adolescents.

## Figures and Tables

**Table 1:** Descriptive statistics of study variables.

	Min	Max	M	SD	95% CI Low	95% CI High
1. Food Insecurity	0	15	4.35	4.19	3.73	4.95
2. Exposure to Violence	2	49	20.03	10.54	18.54	21.56
3. Depression	0	38	9.08	6.72	8.12	10.04
4. Anxiety	0	16	8.33	3.65	7.73	8.95
5. Quality of Life	17	50	38.17	6.49	37.24	39.09
6. Educational Level	0	17	6.44	4.39	5.94	7.33
7. Income per capita	0	2333.33	334.49	392.92	274.35	399.49

**Table 2:** Spearman bivariate correlations.

	1	2	3	4	5	6	7
1. Food Insecurity	1						
2. Exposure to Violence	0.05	1					
3. Depression	−0.10	0.31 ***	1				
4. Anxiety	0.07	0.28 ***	0.51 ***	1			
5. Quality of Life	−0.05	−0.24 **	−0.64 ***	−0.30 ***	1		
6. Educational Level	−0.25 **	−0.04	−0.0	−0.06	0.07	1	
7. Income per capita	−0.41 ***	−0.01	0.24 **	0.08	−0.03	0.48 ***	1

** *p* < 0.01; *** *p* < 0.001

**Table 3:** Differences by demographics in study variables.

		Food Insecurity	Exposure to Violence	Depression	Anxiety	Quality of Life
Gender	Z	0.68	−1.66	−2.75	−0.72	2.35
	Cohen’s d	−0.17	0.24	0.44	0.16	−0.40
	*p*	0.498	0.097	0.006	0.473	0.019
Age	Z	−0.65	1.73	5.93	3.61	−5.30
	Cohen’s d	0.19	−0.16	−1.01	−0.73	0.89
	*p*	0.514	0.084	<0.001	<0.001	<0.001
Habitat	Z	1.02	−5.29	−2.81	0.09	1.63
	Cohen’s d	−0.21	0.89	0.35	0.01	−0.22
	*p*	0.308	<0.001	0.005	0.930	0.103

**Table 4:** Hierarchical regression analysis for depression.

	Depression					
	*t*	β	LLCI	HLCI	*p*	Tolerance
Gender	−1.65	−0.12	−3.35	0.17	0.102	0.95
Age	5.55	0.42	3.35	7.61	<0.001	0.90
Habitat	−0.14	−0.01	−2.65	2.57	0.888	0.77
Educational Level	0.77	0.06	−0.11	0.32	0.444	0.76
Income per capita	1.14	0.10	−0.01	0.01	0.258	0.73
Food Insecurity	0.22	0.02	−0.21	0.26	0.827	0.85
Exposure to Violence	3.11	0.25	0.06	0.26	0.002	0.81

F (7, 134) = 7.98, *p* < 0.001, ΔR^2^ = 0.26.

**Table 5:** Hierarchical regression analysis for anxiety.

	Anxiety					
	*t*	β	LLCI	HLCI	*p*	Tolerance
Gender	−94	−0.09	−2.02	0.64	0.350	0.95
Age	3.20	0.34	0.93	3.94	0.002	0.74
Habitat	1.95	0.21	−0.05	3.18	0.054	0.76
Educational Level	−0.87	−0.09	−0.23	0.08	0.388	0.71
Income per capita	1.86	0.20	0.01	0.02	0.065	0.76
Food Insecurity	0.61	0.06	−0.12	0.20	0.547	0.87
Exposure to Violence	2.00	0.20	0.01	0.17	0.048	0.87

F (7, 96) = 3.28, *p* = 0.004, ΔR^2^ = 0.13

**Table 6:** Hierarchical regression analysis for quality of life.

	Quality of life					
	*t*	β	LLCI	HLCI	*p*	Tolerance
Gender	1.60	0.12	−0.24	3.58	0.112	0.95
Age	−5.07	−0.40	−7.41	−2.91	<0.001	0.90
Habitat	−0.46	−0.04	−3.20	1.94	0.648	0.77
Educational Level	−0.75	−0.06	−0.37	0.14	0.456	0.76
Income per capita	0.26	0.02	−0.01	0.01	0.799	0.73
Food Insecurity	−1.45	−0.12	−0.42	0.04	0.149	0.85
Exposure to Violence	−3.53	−0.29	−0.30	−0.09	0.001	0.81

F (7, 134) = 6.87, *p* < 0.001, ΔR^2^ = 0.23

**Table 7:** Moderation analyses for depression.

	Depression					
Exposure to Violence	F/R^2^	*t*	β	LLCI	HLCI	*p*
X Gender	1.5/0.01	−1.22	−0.09	−0.13	0.02	0.224
X Age	53.5/0.22	7.32	0.48	0.12	0.23	<0.001
X Habitat	6.6/0.03	2.58	0.19	0.01	0.16	0.011
X Educa. Level	6.6/0.03	2.56	0.19	0.01	0.02	0.011
X Income	8.1/0.05	2.85	0.23	0.00	0.01	0.005

**Table 8:** Moderation analyses for anxiety.

	Anxiety					
Exposure to Violence	F/R^2^	*t*	β	LLCI	HLCI	*p*
X Gender	0.01/0.01	0.10	0.01	−0.05	0.05	0.918
X Age	21.8/0.13	4.67	0.37	0.04	0.10	<0.001
X Habitat	10.2/0.06	3.20	0.27	0.02	0.10	0.002
X Educa. Level	3.2/0.02	1.80	0.15	−0.01	0.01	0.075
X Income	3.0/0.02	1.74	.17	−0.01	0.01	0.086

**Table 9:** Moderation analyses for quality of life.

	Quality of Life					
Exposure to Violence	F/R^2^	*t*	β	LLCI	HLCI	*p*
X Gender	1.9/0.01	1.36	0.10	−0.02	0.13	0.176
X Age	36.1/0.16	−6.01	−0.41	−0.19	−0.09	<0.001
X Habitat	7.2/0.03	−2.68	−0.20	−0.15	−0.01	0.008
X Educa. Level	5.1/0.02	−2.25	−0.17	−0.02	−0.01	0.026
X Income	1.4/0.01	−1.18	−0.10	−0.01	0.01	0.239
